# Novelty Seeking and Mental Health in Chinese University Students Before, During, and After the COVID-19 Pandemic Lockdown: A Longitudinal Study

**DOI:** 10.3389/fpsyg.2020.600739

**Published:** 2020-12-03

**Authors:** Wendy Wen Li, Huizhen Yu, Dan J. Miller, Fang Yang, Christopher Rouen

**Affiliations:** ^1^College of Healthcare Sciences, James Cook University, Townsville, QLD, Australia; ^2^Department of Social Work, Foshan University, Foshan, China

**Keywords:** COVID-19, novelty seeking, stress, anxiety, depression, meaning maintenance model, latent growth modeling, Bayesian estimation

## Abstract

COVID-19 has created significant concern surrounding the impact of pandemic lockdown on mental health. While the pandemic lockdown can be distressing, times of crisis can also provide people with the opportunity to think divergently and explore different activities. Novelty seeking, where individuals explore novel and unfamiliarly stimuli and environments, may enhance the creativity of individuals to solve problems in a way that allows them to adjust their emotional responses to stressful situations. This study employs a longitudinal design to investigate changes in novelty seeking and mental health outcomes (namely, stress, anxiety, and depression) before, during, and after COVID-19 pandemic lockdown, among a group of students (final *N* = 173; *M*_age_ = 19.81; *SD*_age_ = 0.98; 135 females and 38 males) from a university in southeast China. Participants were surveyed at three points: November, 2019 (prior to the COVID-19 pandemic); between February and March, 2020 (during the peak of the pandemic and intense lockdown in China); and between May and June, 2020 (after lockdown had been lifted in China). Cross-sectionally, correlation analysis indicated that greater novelty seeking was associated with lower levels of stress, anxiety, and depression at all three time points. Univariate latent curve modeling (LCM) indicated a growth trajectory in which novelty seeking increased over time and then remained high during the post-lockdown period. Stress, anxiety, and depression all showed V-shaped growth trajectories in which these variables decreased during lockdown, before increasing in the post-lockdown period. Multivariate LCM indicated the growth trajectory for novelty seeking was associated with the growth trajectories for stress, anxiety, and depression. This suggests that the observed decreases in stress, anxiety, and depression during the lockdown period may be attributable to the sample’s observed increase in novelty seeking. These findings are valuable in that they challenge the notion that lockdown measures are inherently detrimental to mental health. The findings indicate the important role of novelty seeking in responding to crises. It may be possible for future public health measures to incorporate the promotion of novelty seeking to help individuals’ respond to stressful situations and maintain good mental health in the face of crises.

## Introduction

### Background

The COVID-19 pandemic has presented the world with far reaching and ever-changing challenges that will have widespread and long-term ramifications. The unprecedented spread, drastic public health response, limited treatment options and economic consequences of COVID-19 have created significant concern surrounding both the short- and long-term effects of the pandemic on mental health. Existing literature suggests that the COVID-19 pandemic has led to mental health crises. For example, in a survey investigating the impact of COVID-19 on the mental health of a Chinese cohort, 16.5, 28.8, and 8.1% of participants reported moderate to severe depression, anxiety and stress during the first 2 weeks of the COVID-19 outbreak (Wang et al., 2020). An increasingly globalized and interconnected world greatly heightens the difficulty of containing the virus, which may manifest in more aggravated psychological fear and anxiety compared to previous pandemics, such as SARS in 2003 (Ho et al., 2020).

The concept of existential anxiety and the meaning maintenance model (MMM) offer a theoretical framework for understanding the mental health outcomes resulting from the COVID-19 pandemic. Human beings are meaning-makers, making meaning through building connections, recognizing patterns, and finding associations in scenarios and environments not previously encountered. As meaning-makers, people can establish mental representations of expected relations that connect them with the external world. When some aspects in the external world are not consistent with individuals’ existing and expected relational structures, individuals are likely to develop feelings of the absurd (Camus, 1955; Heine et al., 2006). This feeling of the absurd may lead to psychological distress. The COVID-19 pandemic appears to have disrupted people’s existing and expected relational structures of their families, communities and the world as a whole. The strict lockdown measures, high number of COVID-19 cases and deaths, and extensive media coverage of the virus are likely to have provoked people’s feelings of the absurd, raised awareness of the inevitability of death, and aroused distressing existential anxiety (Pyszczynski et al., 2015). MMM maintains that existential anxiety is not only caused by the fear of death, but also the fear of meaninglessness. The disturbing sense of meaninglessness may motivate people to reconstruct order, normality and certainty, re-establish a sense of coherence, and maintain meaning (Heine et al., 2006) in light of the challenges caused by the COVID-19 pandemic.

According to MMM, meaning is relational and coherently links individuals, community, experience and ideas to one another in expected and predictable ways. People maintain mental health through seeking coherent relations in their everyday lives. As meaning makers, human beings do not passively respond to meaning violations, which involve expected coherent relations and one’s sense of meaning being disrupted by unexpected crisis (Heine et al., 2006; Proulx and Inzlicht, 2012). Instead, people hold a distinctive capacity to detect meaning violations and subsequently reconstruct meaning through re-establishing coherent relations and meaningful associations. Meaning violations caused by the COVID-19 crisis can therefore, on the one hand, result in mental distress, and on the other hand, lead people to reaffirm alternative meaning frameworks so as to maintain mental health. In the process of the reaffirmation of alternative meaning frameworks, MMM posits that fluid compensation is the mechanism that helps people restore meaning following breakdowns. Through fluid compensation, people respond by reaffirming new relational structures to compensate for the damage caused by the meaning violation. This compensatory process is fluid because it does not require a specific relational framework. Any alternative frameworks that are coherent, convincing, and readily available can be employed to compensate for the loss of meaning (Heine et al., 2006; Proulx and Inzlicht, 2012). Through the fluid compensation process, coherent systems of meanings are behaviorally reaffirmed. People may be able to cope with psychological distress caused by the COVID-19 pandemic and even possibly improve their mental health (Proulx and Heine, 2006). Hence, the theory of MMM warrants empirical investigation into changes in mental health before, during, and after the COVID-19 pandemic lockdown.

Meaning maintenance model maintains that there are four domains that are relevant to meaning maintenance: the needs for self-esteem, certainty, belongingness, and symbolic immortality (Heine et al., 2006). These core concepts in meaning maintenance can be reached through creative activities to enhance self-esteem, restore certainty, re-establish belongingness and remind one of life’s joy (Kaufman, 2018). Creativity involves the development of a novel product, idea, or problem solution that is valuable to the individual and/or the larger social group (Hennessey and Amabile, 2010). Creativity can be conceptualized as the capacity to generate novel cognitive content. It is characterized by openness, flexibility, autonomy, playfulness, humor and novelty seeking (Cropley, 1990). These characteristics are important traits that build individuals’ capacity for reaffirming meaning in difficult times (Li, 2010). Such traits may facilitate individuals adapting to the numerous challenges presented by the COVID-19 pandemic.

Novelty is a core component of creativity, and novelty seeking is often a prerequisite to creativity assessment (Jagtap, 2019). As early as in the 1950s, Morgan (1953) reviewed definitions of creativity and concluded that creativity is centered on the concept of novelty. This notion is echoed in the more recent definitions of creativity. For example, Weisberg (1993) defined creativity as novel and useful products and the ability to produce such work. Cropley and Cropley (2005) described creativity in relation to significance, novelty, elegance, and generalizability. Linsey et al. (2008) referred to creativity as the capacity to evaluate problems from different viewpoints, to seek novel possibilities, and produce novelty. Oman et al. (2013) explained creativity as a process to assess a problem in an unexpected or unusual fashion and to produce ideas and work that is novel. Novelty seeking is concerned with people’s propensity to explore novel and new experiences and environments (Arenas and Manzanedo, 2017; Goclowska et al., 2019). Research has established the link between novelty seeking and creativity, where a greater degree of novelty seeking is associated with higher level of creativity in individuals and groups (Goclowska et al., 2019).

There are two broad perspectives on novelty seeking: the neurobiological and the socio-cognitive. Neurobiological studies investigating the relationship between novelty seeking and mental health are largely based on the assessment of risk-taking behavior. The neurobiological approach suggests that novelty seeking behavior is associated with individual differences in specific neurotransmitter activities in the brain (Schweizer, 2006). Novelty seeking is believed to be modulated by the transmission of the neurotransmitter dopamine (Cloninger, 1994), which is determined by specific “novelty-seeking genes” (Prolo and Licinio, 2002). Through analyses of novelty-seeking genes and the brain’s dopamine system, novelty seeking has typically been studied as a risk factor for various behavioral problems concerning risk-taking. From this risk-focused perspective, novelty seeking is conceptualized to have an inhibitory effect on mental health (Ravert et al., 2013). For example, novelty seeking is found to be associated with behavioral problems such as compulsive spending and gambling (Cloninger et al., 1994), and alcohol and substance abuse (Scourfield et al., 1996; Franques et al., 2003). However, Peritogiannis’s (2015) review on novelty seeking in psychotic disorders suggested that novelty seeking was not correlated with suicidality, and that most studies have found no association between novelty seeking and psychiatric patients’ quality of life and mental health outcomes.

Some scholars have noted that neurobiological research may not fully capture the richness of the psychological construct of novelty seeking. They propose that it is problematic to assume that novelty seeking unidimensionally leads to maladaptive and harmful psychological outcomes (Ravert et al., 2013). Instead of focusing on the relationship between novelty seeking and risk behaviors, some researchers adopt a socio-cognitive framework to focus on the association between novelty seeking and various aspects of wellbeing. Socio-cognitive novelty seeking refers to the tendency to have an open and curious orientation to one’s situation and to actively interact with and adapt to the changes in one’s environment (Haigh et al., 2011). Socio-cognitive novelty seeking reflects the social cognitive theory of personality which advocates dynamic dispositions instead of static traits (Bandura, 1999). According to social cognitive theory, dispositions in relation to novelty seeking are personal factors such as self-beliefs, self-construal, and novelty-outcome expectations, which to act regulate novelty seeking behavior. Built upon the concept of information processing, the socio-cognitive approach to novelty seeking emphasizes divergent thinking, producing creative ideas, and exploring various possible solutions, resulting in the production of variability (Cropley, 1990). The production of variability promotes cognitive flexibility, seeking new perspectives (Pirson et al., 2018), performing day-to-day activities differently from the usual, and constructing new meaning of life (Cropley, 1990).

Cognitive flexibility enables the individual to adapt to changing situational demands, reorganize mental resources, construct new perspectives, and balance competing desires and needs (e.g., balancing COVID-19 lockdown compliance with one’s personal desires for autonomy; Haller, 2015). In this way, cognitive flexibility may allow for a cognitive reappraisal of crisis situations, such as the COVID-19 pandemic and its related lockdown measures. Novelty seeking permits individuals to be open to new information and continuously create new categories (Langer, 2014) and representations of conceptual knowledge (Mahon and Caramazza, 2009). Through processing new information, creating new categories and developing new ideas, new meaning can be constructed in a new situation. Consequently, people with high levels of novelty seeking may be better able to evaluate their experiences and situations from multiple perspectives, and use feedback from new situations to modify their behaviors (Haigh et al., 2011).

COVID-19 has resulted in many people being placed into lockdown, leading to social isolation. Novelty seeking may support people to be sensitive to the pandemic context, explore new ideas to overcome isolation, and engage multiple perspectives in coping with stress and anxiety. The awareness of multiple perspectives helps people reaffirm meaning and stimulate compensatory behavior to address the meaning violations caused by the COVID-19 crisis. As such, individuals with high levels of novelty seeking may seek out novelty and new experiences, extend and exercise their capacities, and to learn from new challenges. Novelty seeking thus leads people to encounter the challenges that are optimal for their self-development and reconstruct meaning in difficult times (González-Cutre et al., 2016).

Lockdown may also lead to boredom. When a person is confined to an unstimulating environment for a long time (e.g., a lockdown environment for several months), the person may suffer psychologically. In such an environment, the person is exposed to patterns of stimulation that are perceived as repeated and unvarying (Langer, 2014). Empirical evidence suggests that novelty seeking is negatively associated with boredom (Ritchie and Bryant, 2012). Therefore, the willingness and ability to seek novelty can stimulate the person’s cognitive system and generate positive appraisal.

When individuals have the choice between positive and negative appraisals of a situation, they often choose a positive appraisal (Pirson et al., 2018). As such, novelty seeking may induce positive emotional states. Correlation analyses has provided evidence of the positive association between novelty seeking and psychological wellbeing and mental health. Ravert et al. (2013) found positive correlations between novelty seeking and two wellbeing constructs: eudaimonic wellbeing and psychological wellbeing. Pirson et al. (2018) reported that novelty seeking was positively associated with psychological wellbeing, subjective wellbeing, self-esteem, and life satisfaction; and negatively correlated with negative emotional states. Crescentini et al. (2018) concluded that novelty seeking was positively associated with autonomy, positive relations and personal growth in adolescents.

There is a growing body of scholarly papers on the mental health impacts of COVID-19 (Bao et al., 2020; Brooks et al., 2020; Chen et al., 2020; Dong and Bouey, 2020; Duan and Zhu, 2020; Ho et al., 2020; Lersch, 2020; Pfefferbaum and North, 2020; Pfender, 2020; Pierce et al., 2020; Rajkumar, 2020; Savage et al., 2020; Shah et al., 2020; Steingard, 2020; Usher et al., 2020; Wang et al., 2020; Xiao et al., 2020a,b; Xiong et al., 2020; Yang et al., 2020; Zandifar and Badrfam, 2020; Zhai and Du, 2020; Zhou et al., 2020; Zhu et al., 2020). However, a large proportion of these publications are either commentaries or cross-sectional studies. These cross-sectional studies have found COVID-19 to be associated with a number of negative psychological symptoms including post-traumatic stress symptoms, anxiety, sleeping disturbances, fear of infection, avoidance behavior and anger. Although the cross-sectional studies have be useful in identifying wellbeing variables that may be affected by COVID-19, they have methodological limitations in that they cannot capture changes in participants’ mental health over time or which variables causally contribute to these negative psychological symptoms (Lee and Lee, 2011). To the authors’ knowledge, Pierce et al.’s (2020) and Savage et al.’s (2020) studies are the only longitudinal studies on COVID-19-related distress.

Savage et al.’s (2020) study aimed to investigate the changes in mental health in a sample of 214 students from an East Midlands university in the United Kingdom during the COVID-19 pandemic. The study assessed four points in time—twice before (T1: October 2019 and T2: January 2020) and twice during the lockdown of the United Kingdom (T3: March 2020 and T4: April 2020). The findings of the study showed that during the first 5 weeks of lockdown, participants’ mental wellbeing decreased significantly, while their perceived stress increased significantly. These changes in mental wellbeing and stress were not associated with pre-lockdown mental health.

Savage et al.’s (2020) study is valuable in understanding possible changes in mental health prior and during the COVID-19 pandemic. However, the study made use of repeated-measure ANOVA (Analysis of Variance) which, unlike latent growth modeling (LGM), is not capable of testing individual differences in growth curves or whether growth curves for different variables relate over time (Preacher et al., 2015). LGM allows researchers not only to examine change in an outcome of interest over time (Burant, 2016), but also to investigate intercept and slope variances to evaluate the extent to which individuals change in a given timeframe and the pattern of these changes. Multiple intercepts and slopes can be covaried to investigate how changes in one variable (e.g., novelty seeking in the present study) relate to changes in other variables (e.g., stress, anxiety and depression in the present study; Stull, 2008).

In the secondary analysis of cohort data from the United Kingdom Household Longitudinal Study (UKHLS), Pierce et al. (2020) investigated changes in adult mental health in the United Kingdom population before and during the COVID-19 lockdown. The findings indicated that population prevalence of clinically significant levels of mental distress rose from 18.9% in 2018–2019 to 27.3% in April 2020, 4 weeks into the United Kingdom COVID-19 lockdown. The mean score for the General Health Questionnaire (GHQ-12) also increased over the period, indicating the levels of mental distress increased. The greatest increases were observed in 18–24-year-olds, 25–34-year-olds, women, and people living with young children. The findings also reported that the higher increase in mental distress did not affect all groups equally. People in some demographic groups (e.g., those living with a partner, having an underlying health condition, being an essential worker, and being unemployed before COVID-19) showed little (or no) additional mental distress during the lockdown. This finding is meaningful in that it suggests that while many people have experienced increased mental distress during the COVID-19 pandemic, some people may be able to maintain pre-COVID-19 levels of mental wellbeing.

### The Current Study

Employing a longitudinal design, this exploratory study aims to extend research on mental health during the COVID-19 pandemic through the assessment of novelty seeking and mental health among a sample of Chinese university students, over a 6-month period. As the first longitudinal investigation on the association between novelty seeking and mental health during the COVID-19 crisis, the present study has the potential to offer information that could be utilized in public health campaigns to minimize the negative mental health effects of pandemics and lockdown measures. Three points in time are assessed: prior to the COVID-19 crisis (T1), during COVID-19 lockdown (T2), and after the COVID-19 pandemic was relatively well-controlled within China and lockdown measures had been lifted (T3). Similar to Savage et al.’s (2020) study, the present study incorporates a time point before the pandemic outbreak.

Built upon the previously reviewed evidence of the association between novelty seeking and mental health, the current study hypothesizes that novelty seeking, stress, anxiety and depression will be associated with one another at each time point (H1). To examine change in novelty seeking, stress, anxiety and depression over time, two research questions are investigated: (1) What is the average growth trajectory for novelty seeking, stress, anxiety, and depression across the three time points, and are there substantial individual differences in these growth trajectories? (2) Do the growth trajectories for stress, depression, and anxiety relate to the growth trajectory for novelty seeking?

## Materials and Methods

### Participants

Participants were recruited at a university in a southeast city of China to participate in a longitudinal study that aimed to investigate mental health among second- and third-year students across five disciplines. The five disciplines were: social work, international economics, economics and trade, marketing, and accounting. An email was sent in September 2019 to all second- and third-year students in the five disciplines (*N* = 619) to invite them to take part in the study. A total of 203 students participated in the T1 survey, resulting in a response rate of 32.8%. Of these 203 participants, 15 participants did not take part in the T2 and T3 surveys and 10 did not participant in T2 survey only. These 25 participants were removed, resulting in a sample of 178 participants. At T1, all students resided in the university’s host city. At T2 and T3, the majority of participants resided in Guangdong Province (*N* = 157, 88.2%) where there were 1,579 confirmed cases of COVID-19 and eight deaths as of April 17, 2020 (Health Commission of Guangdong Province, 2020). The remaining students (*N* = 21, 11.8%) resided in Hainan, Hunan, Henan, Jiangxi, and Yunnan Provinces, where the COVID-19 infection rates were lower than that in Guangdong Province. There were 137 females (77%) and 41 males (23%), with an average age of 19.8 years (range = 18–22, *SD* = 0.97). There were 46, 25, 23, 48, and 36 participants in the disciplines of social work, international economics, economics and trade, marketing, and accounting, respectively.

### Measures

#### Novelty Seeking

Novelty seeking was measured using the 5-item novelty seeking subscale of the 14-item Langer Mindfulness Scale (LMS; Pirson et al., 2018). The five items measuring novelty seeking are “I like to investigate things,” “I am very curious,” “I try to think of new ways of doing things,” “I like to be challenged intellectually,” and “I like to figure out how things work.” Participants were asked to rate each item using 7-point Likert-type scales (where 1 = *Strongly disagree* and 7 = *Strongly agree*). Items were summed to produce a total sore, where higher values correspond to greater novelty seeking.

#### Stress, Anxiety and Depression

The standardized Chinese version of the short Depression Anxiety Stress Scale (C-DASS21; Taouk et al., 2001) was employed to measure past-month stress, anxiety and depression. The 21-item short DASS is a self-report scale with 7 items each for depression, anxiety, and stress. Sample items include “I was aware of dryness of my mouth,” “I couldn’t seem to experience any positive feeling at all” and “I experienced breathing difficulty (e.g., excessively rapid breathing, breathlessness in the absence of physical exertion).” All items are on a 4-point scale (where 0 = *Did not apply to me at all* and 3 = *Applied to me very much, or most of the time*). Items were summed to produce total scores for stress, anxiety, and depression, where higher scores indicate greater depression, anxiety and stress. As suggested by the scale authors, subscale scores were multiplied by two to be consistent with the 42-item DASS (Taouk et al., 2001).

### Internal Reliability Estimates

Internal reliability estimates for the measures used in the present study are provided in Table 1. The results showed that all measures had good internal reliability.

**TABLE 1:** Cronbach’s alphas based on the current and source studies.

Instrument	T1	T2	T3	Source study
Novelty Seeking	0.74	0.87	0.89	0.75–0.86 (Pirson et al., 2018)
Stress	0.77	0.86	0.85	0.70 (Oei et al., 2013)
Anxiety	0.70	0.82	0.82	0.87 (Xie et al., 2020)
Depression	0.80	0.86	0.84	0.88 (Xie et al., 2020)

### Procedure

Ethical approval for the study was obtained from the Human Research Ethics Committee of the Department of Social Work, Foshan University, China (Ref. 2019001). A paper-and-pencil survey was administrated at T1, while online surveys were conducted via wenjuanxin.cn at T2 and T3. The interval between each point in time was 3 months. T1 data was collected between November 20 and 28, 2019—prior to the COVID-19 crisis. The T2 survey was administered from February 28 to March 10, 2020—during the COVID-19 outbreak and lockdown. Finally, the T3 survey was conducted between May 29 and June 10, 2020—after the lockdown was lifted and COVID-19 relatively contained in China. Each participant received RMB¥8.88 (equivalent to USD$1.25 at the time of data collection) at T3.

### Data Cleaning

Inspection of missing values indicated that missing data were minimal, with almost all scale items having no missing responses and two scale items missing one response only. Expectation maximization was used to estimate values for these two missing data points.

As outliers can produce non-admissible results in structural equation modeling (especially when sample sizes are relatively small; Kline, 2016) variables were assessed for univariate outliers using a combination of graphical inspection (histograms and boxplots) as well as the outlier labeling rule with a 2.2 multiplier (Hoaglin and Iglewicz, 1987). To retain as much of the sample as possible, identified univariate outliers were assigned the next highest observed, non-outlier value +1 (to maintain the rank order of the data). Seven such univariate outliers were identified.

Mahalanobis distance figures were used to screen for multivariate outliers. Five multivariate outliers were detected using an α of 0.001 (Tabachnick and Fiddel, 2013) and deleted. This left a final *N* of 173.

### Analysis

Correlation analysis was used to test H1. LGM was used to investigate RQs 1 and 2. In LGM, models produce estimates of intra-individual change and inter-individual differences in these change parameters. Intra-individual change estimates include the average starting value for the outcome of interest (the intercept mean) and the average growth trajectory in the outcome of interest (the slope mean). Intercept and slope variances indicate the degree of individual-difference in these growth processes. The intercept and slope variances represent the degree of individual difference in the starting value and growth trajectory of the outcome, respectively.

Univariate LGM involves modeling the growth trajectory of a single outcome. However, it is also possible to perform multivariate LGM in which multiple growth trajectories are estimated in the same model, in order to assess how two or more variables change together over time (Bollen and Curran, 2006; Kaplan, 2009).

RQ1 was investigated using four univariate LCMs, one for each outcome variable. RQ2 was investigated via three multivariate LCMs: one for novelty seeking and stress; novelty seeking and anxiety; and novelty seeking and depression. Three separate models were employed to avoid issues of multicollinearity. It is common practice to perform univariate LCM before adding additional variables, in order to better identify sources of poor model fit (Kline, 2016).

All LCMs were constructed in Amos version 25. In the current study, construction of the univariate LCMs involved treating each time point measure as an observed variable. Two latent factors were then constructed, one to represent the intercept of the outcome of interest and one to represent the slope of the outcome of interest. These latent factors were then freed to covary. Error term variances were constrained to be equal and error term means were fixed to zero. Error terms were not freed to covary. Paths from the intercept factor to the three time point measures were all fixed to 1. Paths from the slope factor to the three time point measures were fixed to 0, 1, and 2, respectively. Given that these weights are evenly spaced, this specifies a linear growth in the variable (Kline, 2016).

It is possible to model other forms of growth in addition to linear growth, for example, quadratic growth (see Burant, 2016). However, there are practical issues with modeling quadratic growth when data have been collected at fewer than four points in time (Henry and Slater, 2008), especially in smaller samples. For example, Diallo et al. (2014) indicate that quadratic growth modeling with three time points increases the rate of model non-convergence. They propose that a sample size of at least 250, with four measurement points, is required in order to capture quadratic change. It is also possible to fit a non-linear growth curve (Kaplan, 2009; Kline, 2016). This is done by fixing the loadings from the slope factor to the first and second time point measures to 0 and 1, while allowing the remaining loadings between the slope factor and time point measures to be freely estimated. Accordingly, in the current study, if a linear growth trajectory was found to produce a poor fitting model, a non-linear growth trajectory was then modeled.

In multivariate LGM, intercept and slope factors for each variable are freed to covary. This allows for the examination of cross-domain intercept-slope covariances (which indicates whether the starting value in one factor is associated with the rate of change in the other), intercept-intercept covariances (which indicates whether the starting value in one variable is associated with the starting value in the other), and most importantly, slope-slope covariances (which indicates whether the rate of change in one variable is associated with the rate of change in another, i.e., whether the two variables *travel together through time*, Bollen and Curran, 2006).

## Results

### Test of H1

Table 2 presents means and standard deviations for the study variables, as well as correlations between all study variables across T1, T2, and T3. Age and gender are also included in the analysis. There were moderate to strong positive correlations between the three time point measures for novelty seeking (*r* = 0.54–0.70), stress (*r* = 0.51–0.70), anxiety (*r* = 0.52–0.62), and depression (*r* = 0.51–0.71).

**TABLE 2:** Pearson correlations between study variables and means (standard deviations) across the three time points.

	T1 NS	T2 NS	T3 NS	T1 Stress	T2 Stress	T3 Stress	T1 Anx	T2 Anx	T3 Anx	T1 Dep	T2 Dep	T3 Dep	Age	Gender	Mean (*SD*)
T1 NS	–	0.54***	0.56***	−0.16*	–0.12	–0.08	−0.21**	–0.08	–0.13	−0.26***	−0.17*	−0.17*	–0.03	0.01	24.47 (4.66)
T2 NS		–	0.70***	−0.17*	−0.28***	−0.30***	−0.20**	−0.28***	−0.27***	−0.26**	−0.33***	−0.38***	–0.04	–0.04	25.80 (4.77)
T3 NS			–	–0.13	−0.28***	−0.26***	−0.23**	−0.29***	−0.30***	−0.22**	−0.37***	−0.37***	–0.03	0.02	25.65 (5.05)
T1 Stress				–	0.52***	0.51***	0.75***	0.51***	0.46***	0.64***	0.46***	0.51***	0.13	–0.10	10.95 (7.05)
T2 Stress					–	0.69***	0.50***	0.83***	0.58***	0.42***	0.80***	0.63***	0.19*	–0.10	7.11 (7.18)
T3 Stress						–	0.50***	0.60***	0.80***	0.46***	0.57***	0.79***	0.17*	–0.03	8.77 (7.04)
T1 Anx							–	0.58***	0.52***	0.68***	0.50***	0.57***	0.07	–0.06	9.23 (6.16)
T2 Anx								–	0.62***	0.41***	0.82***	0.64***	0.13	–0.05	5.09 (5.90)
T3 Anx									–	0.42***	0.61***	0.80***	0.03	–0.08	6.74 (5.97)
T1 Dep										–	0.51***	0.61***	0.05	–0.06	6.25 (6.15)
T2 Dep											–	0.71***	0.12	–0.08	4.99 (6.15)
T3 Dep												–	0.06	–0.05	6.01 (6.10)
Age													−	0.05	19.81 (0.98)
Gender														–	NA

*p < 0.05; **p < 0.01; ***p < 0.001. All significance tests are two-tailed; df = 171 for all tests; correlations in gender column are point-biserial correlations. NS, Novelty Seeking; Dep, Depression; Anx, Anxiety.

Within each time point, total scores for stress, anxiety, and depression were all highly positively correlated (for T1 *r* = 0.64–0.75; for T2 *r* = 0.80–0.83; for T3 *r* = 0.79–0.80), as would be expected given the conceptual overlap in these constructs. T1 novelty seeking showed small (albeit significant) negative correlations with T1 stress, anxiety, and depression (*r* = −0.16 to −0.26). T2 novelty seeking showed small to moderate negative correlations with T2 stress, anxiety, and depression (*r* = −0.28 to −0.33), as did T3 novelty seeking with T3 stress, anxiety, and depression (*r* = −0.27 to −0.36).

As outlined in Table 2, T2 novelty seeking was a significant negative predictor of stress, anxiety, and depression at all three points in time, as was T3 novelty seeking (with the exception of T1 stress). Conversely, T1 novelty seeking was a negative predictor of T1 anxiety, T1 stress, and T1, T2, and T3 depression, but not T2 and T3 anxiety or T2 and T3 stress. Together these results show support for H1.

### Test of RQ1

#### Novelty Seeking

**FIGURE 1:**
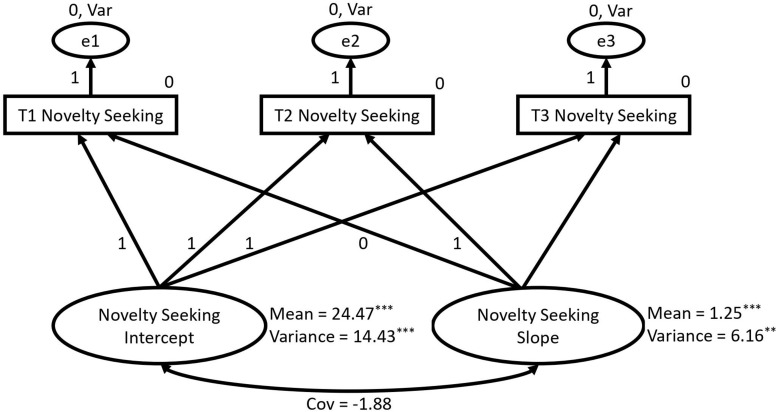
Univariate latent curve model specifying a non-linear growth trajectory for novelty seeking (^∗∗^*p* < 0.01; ^∗∗∗^*p* < 0.001). Pathways fixed to 0 or 1 were constrained in order to model non-linear growth (see section *“*Analysis”). Error term variances were constrained to be equal and error term means were fixed to 0. Cov, covariance.

A univariate LCM was constructed in which a linear growth curve was specified for novelty seeking and model fit was assessed, χ^2^ = 13.85, *df* = 3, *p* = 0.003, CFI = 0.94, RMSEA = 0.145, SRMR = 0.033. The linear model showed reasonable fit according to some fit indices (CFI and SRMR), but not others (χ^2^ test and RMSEA). Accordingly, the factor loading from the slope factor to T3 novelty seeking was freely estimated to model non-linear growth. This non-linear model showed good fit, χ^2^ = 1.49, *df* = 2, *p* = 0.475, CFI > 0.99, RMSEA < 0.001, SRMR = 0.006. The unstandardized loading between the slope factor and T3 novelty seeking was estimated at 0.99. The retained model is depicted in Figure 1.

Means and variances for the intercept and slope factors for all univariate models are reported in Table 3. The slope factor mean was significant, indicating non-linear growth in novelty seeking across the three time points. The variance of the slope factor was significant, suggesting individual differences in this growth trajectory. The intercept factor mean indicates the average starting value in novelty seeking (24.47 in this case), and the significance of the intercept variance indicates substantial individual difference in starting levels of novelty seeking. The covariance between the intercept and slope factors was non-significant, indicating that level of novelty seeking at T1 had little impact on the rate of growth in novelty seeking.

**TABLE 3:** Means and variances for intercept and slope factors, as well as covariances between intercept and slope factors, for all univariate latent growth curve models.

Model	Mean of Intercept	Variance of Intercept	Mean of Slope	Variance of Slope	Covariance
Novelty Seeking	24.47***	14.43***	1.25***	6.16**	–1.88
Stress	11.05***	31.86***	−3.70***	18.18**	–6.47
Anxiety	9.24***	21.65***	−4.13***	2.25	–1.44
Depression	6.38***	27.24***	−1.15*	15.83***	−7.19*

*p < 0.05; **p < 0.01; ***p < 0.001; for all variables, non-linear growth is being modeled.

**FIGURE 2:**
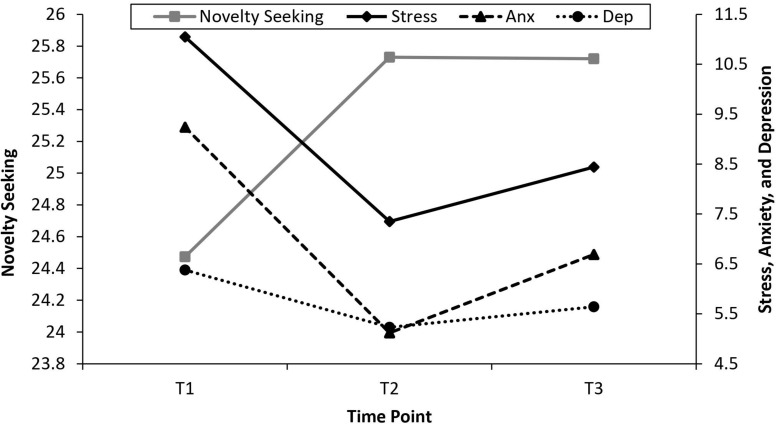
Growth trajectory for novelty seeking, stress, anxiety, and depression. Model-implied means (generated as part of section “Test of RQ1”) used in plot.

Burant (2016, p. 345) provides a formula for calculating model-implied means for growth models: model-implied mean = mean of intercept + [mean of slope × unstandardized factor loading at time (x)]. Using this formula produces model-implied means of 24.47, 25.73, and 25.72 at T1, T2, and T3, respectively. This indicates a pattern of growth in which novelty seeking increased between T1 and T2 and then “leveled-off” between T2 and T3. Figure 2 uses the model-implied means derived from the univariate LCMs to plot the prototypical growth trajectories for novelty seeking, stress, anxiety, and depression. These growth trajectories are superimposed to assist in visualizing how the growth trajectories traveled together over the study period.

#### Stress

A LCM specifying a linear growth trajectory in stress was not a good fit to the data, χ^2^ = 52.72, *df* = 3, *p* < 0.001; CFI = 0.72, RMSEA = 0.310, SRMR = 0.026. Accordingly, non-linear growth was modeled resulting in good model fit, χ^2^ = 5.83, *df* = 2, *p* = 0.054; CFI = 0.98, RMSEA = 0.106, SRMR = 0.057. The unstandardized loading between the slope factor and T3 stress was estimated at 0.70. The retained model is identical in structure to the one depicted in Figure 1 (as are the models produced for anxiety and depression).

The significance of the slope mean indicates non-linear growth in stress across the study period. The slope variance was significant, suggesting individual difference in this growth trajectory. Furthermore, the intercept variance was significant, indicating individual difference in the average starting value for stress. The covariance between the intercept and slope factor was non-significant, suggesting that starting levels of stress were not associated with the rate of growth in stress. As opposed to the growth trajectory for novelty seeking, the model-implied means indicate that stress decreased from T1 (11.05) to T2 (7.35), before increasing somewhat at T3 (8.44).

#### Anxiety

The anxiety data similarly did not fit a linear growth trajectory, χ^2^ = 61.27, *df* = 3, *p* < 0.001; CFI = 0.64, RMSEA = 0.336, SRMR = 0.052. However, a non-linear growth trajectory showed good fit: χ^2^ = 4.00, *df* = 2, *p* = 0.135; CFI = 0.99, RMSEA = 0.076, SRMR = 0.028. The unstandardized loading between the slope factor and T3 anxiety was estimated at 0.62.

Once again, significant non-linear growth was observed. The non-significance of the slope variance suggests minimal individual difference in this growth trajectory. Whereas, there appears to be substantial individual differences in initial levels of anxiety, as indicated by the significance intercept variance. As above, the covariance between the intercept and slope factor was non-significant. The model-implied means showed a similar growth trajectory to stress, with anxiety decreasing from T1 (9.24) to T2 (5.12), before increasing at T3 (6.69).

#### Depression

Specifying a linear growth trajectory in depression resulted in poor model fit, χ^2^ = 25.32, *df* = 3, *p* < 0.001; CFI = 0.89, RMSEA = 0.147, SRMR = 0.050, whereas a non-linear growth trajectory was found to fit the data well, χ^2^ = 4.77, *df* = 2, *p* = 0.092; CFI = 0.99, RMSEA = 0.090, SRMR = 0.009. The unstandardized loading between the slope factor and T3 depression was estimated at 0.64.

A significant non-linear growth trajectory was observed, with significant individual variations around this growth trajectory. Individual differences were also observed around the average initial starting value in depression. The significant covariance between the intercept and slope factors indicates that initial levels of depression were associated with the rate of change in depression over time. Once again, the model-implied means indicate a V-shaped growth trajectory (T1 = 6.38, T2 = 5.23, T3 = 5.64).

### Test of RQ2

#### Novelty Seeking and Stress

**FIGURE 3:**
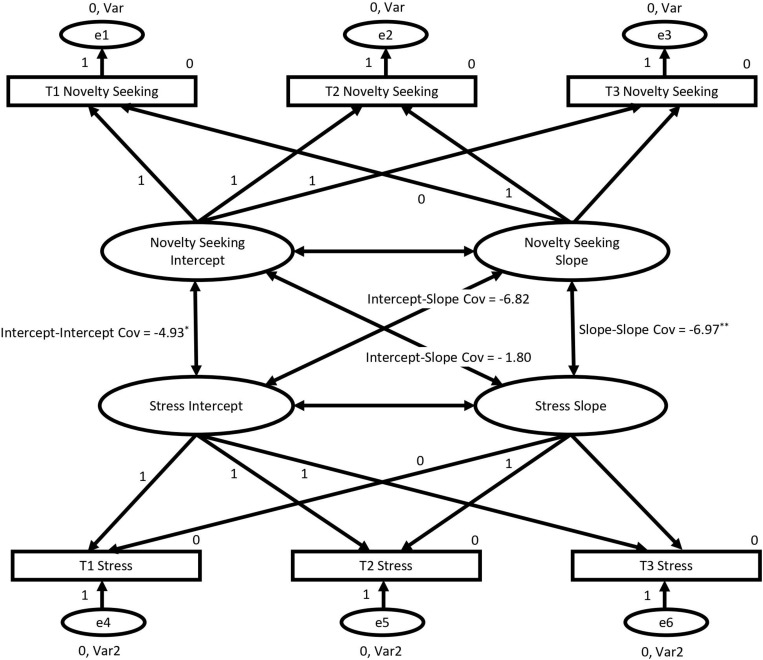
Multivariate latent curve model assessing the relationship between the growth trajectories for novelty seeking and stress, with cross-domain covariances provided (^∗^*p* < 0.05; ^∗∗^*p* < 0.01). Pathways fixed to 0 or 1 were constrained in order to model non-linear growth. Error term variances were constrained to be equal among each variable and error term means were fixed to 0. Cov, covariance.

A multivariate LCM was constructed to assess whether the growth trajectories for novelty seeking and stress were associated. This model showed good fit: χ^2^ = 10.62, *df* = 9, *p* = 0.302; CFI > 0.99, RMSEA = 0.032, SRMR = 0.019. The model is depicted in Figure 3.

The cross-domain covariances for the multivariate LCM for novelty seeking and stress are reported in Table 4. Importantly, the covariance between the slope factor for novelty seeking and the slope factor for stress (the cross-domain slope-slope covariance) was significant. This indicates that the growth trajectory for novelty seeking was associated with the growth trajectory for stress. The only other cross-domain covariance to be significant was between the intercept for novelty seeking and the intercept for stress (the cross-domain intercept-intercept covariance). This relationship was negative, indicating that higher starting values for novelty seeking were associated with lower starting values for stress, and vice versa.

**TABLE 4:** Cross-domain covariances for multivariate growth curve model for novelty seeking and stress and novelty seeking and depression.

Covariance Relationship Being Assessed	Covariance Value
**Novelty Seeking and Stress Model**	
Novelty Seeking Slope – Stress Intercept	−6.82
Novelty Seeking Intercept – Stress Slope	1.80
Novelty Seeking Intercept – Stress Intercept	−4.93*
Novelty Seeking Slope – Stress Slope	−6.97**
**Novelty Seeking and Depression Model**	
Novelty Seeking Slope – Depression Intercept	0.03
Novelty Seeking Intercept – Depression Slope	2.88
Novelty Seeking Intercept – Depression Intercept	−7.41***
Novelty Seeking Slope – Depression Slope	−6.47***

*p < 0.05; **p < 0.01; ***p < 0.001.

#### Novelty Seeking and Anxiety

The multivariate LCM for novelty seeking and anxiety (structurally equivalent to the one depicted in Figure 3) displayed good fit, χ^2^ = 5.96, *df* = 9, *p* = 0.744; CFI > 0.99, RMSEA < 0.001, SRMR = 0.018. However, it produced a non-positive definitive covariance matrix, making the solution unreliable. Bayesian estimation can eliminate the kinds of inadmissible parameters which lead to non-positive definitive solutions (van de Shoot et al., 2014). Accordingly, the model was re-run using Bayesian estimation with diffuse priors. The model converged, with the highest global convergence statistic (1.0016) falling below the critical value of 1.002 (Arbuckle, 2017, p. 415) at around the 40,000th sample. The posteriori predictive *p*-value was 0.59—far above the 0.10 cut-off recommended by Cain and Zhang (2019)—suggesting good model fit.

Table 5 outlines mean covariances between intercept and slope factors in the model, along with their 95% credibility intervals. In lieu of *p*-values, credibility intervals being entirely above or below zero were used to identify statistical significance. As can be seen from the table, the cross-domain slope-slope covariance was significant, indicating that the growth trajectory for novelty seeking was related to the growth trajectory for anxiety. The intercept–intercept covariance was significant and negative, indicating the higher starting values in novelty seeking were associated with lower starting values in anxiety.

**TABLE 5:** Cross-domain mean covariances (and 95% Credibility Intervals) for Bayesian-estimated, multivariate growth curve model for novelty seeking and anxiety.

Covariance Relationship Being Assessed	Mean Covariance Value [LL 95% CI, UL 95% CI]
Novelty Seeking Slope – Anxiety Intercept	−0.79 [−5.11, 3.26]
Novelty Seeking Intercept – Anxiety Slope	3.99 [−0.12, 8.55]
Novelty Seeking Intercept – Anxiety Intercept	−6.36 [−11.43, −1.89]*
Novelty Seeking Slope – Anxiety Slope	−6.08 [−10.54, −2.32]*

*Credibility interval does not contain zero.

*LL 95% CI, lower limit 95% credibility interval; UL 95% CI, upper limit 95% credibility interval.*

#### Novelty Seeking and Depression

A multivariate LCM was constructed for novelty seeking and depression. This model showed good fit: χ^2^ = 11.68, *df* = 9, *p* = 0.232; CFI = 0.99, RMSEA = 0.042, SRMR = 0.015.

The cross-domain slope-slope covariance was significant, indicating that the growth trajectories for novelty seeking and depression were associated. Once again, the intercept-intercept covariance was significant, meaning that higher starting values in novelty seeking were associated with lower starting values in depression, and vice versa. No other cross-domain covariance was significant.

## Discussion

### General Discussion

The current exploratory study used a sample of Chinese university students to investigate growth trajectories in novelty seeking, stress, anxiety, and depression from before (T1), to during (T2), and after (T3), a COVID-19 lockdown. The study also sought to assess whether novelty seeking would change in parallel to stress, anxiety, and depression.

Interestingly, the latent curve modeling indicated a significant growth trajectory in which novelty seeking increased going from pre-COVID-19 to the lockdown period, and then remained relatively stable into the post-lockdown period. Conversely, stress, anxiety, and depression all showed a V-shaped pattern of growth, in which the nadir of these variables was during the lockdown period, with a slight increase post-lockdown. To the authors’ best knowledge, the results of the present study are the first to provide longitudinal evidence of a significant decrease in stress, anxiety, and depression during COVID-19 lockdown. The current study provides a nuanced contrast to the bulk of existing scientific literature (Bao et al., 2020; Brooks et al., 2020; Chen et al., 2020; Dong and Bouey, 2020; Duan and Zhu, 2020; Ho et al., 2020; Lersch, 2020; Pfefferbaum and North, 2020; Pfender, 2020; Pierce et al., 2020; Rajkumar, 2020; Savage et al., 2020; Shah et al., 2020; Steingard, 2020; Usher et al., 2020; Wang et al., 2020; Xiao et al., 2020a,b; Xiong et al., 2020; Yang et al., 2020; Zandifar and Badrfam, 2020; Zhai and Du, 2020; Zhou et al., 2020; Zhu et al., 2020) which generally highlights the highly stressful nature of the COVID-19 pandemic and associated lockdown measures. This is also inconsistent with the findings of Pierce et al.’s (2020) and Savage et al.’s (2020) longitudinal studies, which reported that mental health indicators decreased during the COVID-19 lockdown in the United Kingdom. The findings in the current study suggest that some groups may maintain (or even improve) mental health during lockdown.

There are several possible factors which may have contributed to the improvements in mental health that were observed. First, the decrease in stress, anxiety and depression may have been driven by increases in novelty seeking. Cross-sectionally, novelty seeking displayed a significant negative relationship with stress, anxiety, and depression at each time point. Longitudinally, multivariate growth curve modeling found that the growth trajectory for novelty seeking was associated with the growth trajectories for stress, anxiety, and depression. That is, the mental health variables were found to travel together with novelty seeking. The plot of the prototypical growth trajectories for each of these variables indicated an inverse relationship. When novelty seeking was at its peak, stress, anxiety, and depression were at their nadir. Taken together, these results provide support for the notion that novelty seeking can contribute to improvements in mental health in the context of a quarantine pandemic.

According to MMM, novelty seeking in this case may function as fluid compensation to support individuals in reaffirming new meaning, thus compensating for damage caused by the meaning violations in the COVID-19 lockdown. For example, novelty seeking may promote participants to evaluate and adapt the changing situational demands associated with the pandemic, reconfigure their mental resources and capacities (e.g., being more creative to overcome boredom), thereby increasing cognitive flexibility and generating positive appraisals and emotion (Pirson et al., 2018). Through this process, participants are likely to improve self-esteem, re-establish certainty and re-construct a sense of belonging (Kaufman, 2018), leading to the enhancement of mental health.

Second, alternative academic arrangements in response to COVID-19 lockdown may have also contributed to the observed improvements in mental health. Student life during the COVID-19 lockdown may be less stressful for some students. T1 data were collected during an academic semester where students experienced pressure associated with assignment submission deadlines, fear of failure, studying fatigue, lack of choice in everyday life, meeting social expectations and competing against peers (Langer, 2014). T2 and T3 data were collected when participants stayed home with their parents in mildly infected areas. During the period, participants may have felt less stress as they could have received more support from their parents and the university. The disruptions to participants’ daily schedules may have also provoked participants to consider their lives and social environment in new ways, such as performing self-reflection exercises (e.g., yoga), engaging in art and music activities (e.g., learning painting and playing a new musical instrument through online programs) and learning new skills (e.g., gardening, baking and video making). These novelty seeking activities may help people create new categories, develop new ideas and perspectives (Pirson et al., 2018), and actively adapt to the changing environment (Haigh et al., 2011), therefore reaffirming meaning in a way which decreases psychological distress, as suggested by MMM (Proulx and Heine, 2006).

Third, some cultural factors may have contributed to participants’ reconstruction of connections and a sense of coherence. For example, in Chinese culture, filial piety specifies moral norms (Bedford and Yeh, 2019) around the child’s obligations to defer to their parents’ wishes and obey their parents (Li et al., 2010; Li and He, 2019). Filial piety extends beyond the household to the nation state. This is evident in Chinese popular culture. For example, the motherland (China) being a “dear mother” is a common motif in Chinese music, reinforcing the social expectation of being loyal to authority for Chinese people (Li, 2013). Filial piety moralizes the relationship between government and citizens. It promotes the notion that those in authority tend to make decisions consistent with the principles of righteousness, kindness, and benevolence, and that citizens are expected to demonstrate loyalty, submission, and obedience in exchange (Hwang, 1999). Accordingly, participants may have believed that the quarantine measures introduced by the government were righteous, kind, and benevolent, and for the purpose of protecting them from harm. This trust in government may have resulted in participants being more willing to make self-sacrifices (e.g., complying with lockdown requirements) for the collective good; viewing the lockdown as the “right” thing. In this way, complying with lockdown restrictions is entirely congruent with the cultural belief that Chinese people ought to be responsible selves who suppress their personal wishes and desires to perform their duty to their country. All these factors may have worked to mitigate participants’ anxiety and bolster their collective identity.

Participants’ willingness to comply with enforced lockdowns, may have resulted in less conflicts between the lockdown and a personal desire for freedom (Haller, 2015) and more importantly the establishment of new, coherent and strong connections with their “metaphorical parent,” thereby creating new relational structures in their meaning system. This reflects MMM’s position that people are able to detect meaning violations and reaffirming meaning through re-constructing a coherent relational structure and meaningful associations (Heine et al., 2006; Proulx and Inzlicht, 2012). It also mirrors MMM’s notion that people not only respond to a relational anomaly through reinterpretation of the crisis event or review of the existing relations, but also respond by reaffirming new relational structures to compensate for destruction of the relational framework weakened by the anomaly (Heine et al., 2006).

Fourth, the young age of the participants may contribute to the maintenance of mental health. Participants would likely be aware of the relatively lower risk of becoming seriously ill if they contracted COVID-19 compared to older age groups. This is reflected in Commodari and La Rosa’s (2020) study where young Italians believed that COVID-19 did not pose a serious danger to them. The authors propose that the Italian adolescents’ negative psychological states during the COVID-19 quarantine might have been more related to the adolescent period than to the pandemic itself.

Additionally, cognitive risk perception may also play a role in the improvement of mental health among the students. Existing research suggests that health risk perception impacts people’s mental health (e.g., depression) and psychological wellbeing (e.g., self-efficacy, vulnerability) during public health crises (Commodari, 2017; Commodari et al., 2020; Ding et al., 2020). Ding et al. (2020) found that in a sample of Chinese participants, cognitive health risk perception was negatively associated with depression during the COVID-19 pandemic. Cognitive health risk perception is a form of information processing that is cautious, controllable and rational, which may help people remain calm in times of crisis. Research has found that novelty seeking is positively associated with risk perception (Chang, 2011). It is possible that higher scores of socio-cognitive novelty seeking at T2 and T3 may have increases participants’ cognitive risk perception, which in turn contributed to the increases in mental health.

The current findings also show significant individual differences in stress, anxiety and depression at T1 and in the growth curves of stress and depression. These differences may be explained by MMM’s proposition that the need for maintaining a coherent relational structure is a fundamental motivation that leads people to cope with anomaly in crisis circumstances (Heine et al., 2006). Individuals are likely to have differences in the need for new relational structures. Such individual differences may influence how individuals comprehend, involve, and interact with the changed world in the time of COVID-19. According to Neuberg and Newsom (1993), some people possess a tendency to create and maintain simple cognitive structures for constructing relations, indicating their general non-complexity and preference for routine. Individuals with high levels of need for simple cognitive structure are particularly sensitive to changing events and may be especially emotionally reactive (Neuberg and Newsom, 1993). They may experience greater levels of emotional reactions to anomaly, such as those caused by COVID-19. Individuals who tend to maintain more complicated cognitive structures for building relations are likely to reduce uncertainty in crisis situations (Svecova and Pavlovicova, 2016), which is associated with a greater ability to meet the needs of the new situation.

The consideration of individual differences in the analysis may have contributed to the differences in the results of this study compared to other similar studies. For example, Savage et al.’s (2020) study used repeated-measure ANOVA, which fails to test for individual differences. Moreover, the different measurement instruments utilized between studies may also contribute to different results.

Finally, the degree to which stress, anxiety, and depression were positively related is also worth acknowledging. The high correlations between these variables observed at each time point (see Table 2) emphasizes how closely linked these constructs are.

### Practical Implications

Despite these limitations, the findings highlight some of the potential benefits of giving individuals the space to engage in novelty seeking activities. First, the results of this study tentatively suggest that novelty seeking activities not only help individuals to maintain mental health in the face of adversity, but may even allow them to thrive in such situations. Parallels can be drawn between the experiences of this student sample and many white-collar workers (e.g., shifting from a highly regimented on-campus mode of learning to a digitally connected, but physically distanced, method of learning). This raises questions as to whether providing employees more flexibility in their mode of work, outside of the COVID-19 context, would result in similar gains in novelty seeking and mental wellbeing.

Second, the current findings provide relevant information for designing and implementing mental health programs for COVID-19 and future pandemics. The present analysis suggests that although the awareness of death often generates negative outcomes, it can also function to move people along more positive directions and contribute to their mental health (Vail et al., 2012). When people function securely and have strong faith that they are significant contributors to the battle against COVID-19, they are likely to build supportive relationships, maintain mental health, and foster open-minded and growth-oriented behaviors. This has important ramifications for public health strategies during infectious disease outbreaks. The inclusion of strategies that buttress psychological resources such as novelty seeking (and creativity more broadly) may assist in mitigating some of the more negative effects of lockdown.

Third, the results of the study undercut the notion that lockdown measures automatically result in poorly mental health. Existing literature on mental health and COVID-19 is decidedly negative in focus and places emphasis on deficits in human functioning. The current study provides a more positive perspective, where novelty seeking functions as a contributing factor that allows people to maintain feelings of wellbeing, when facing the COVID-19 crisis.

### Limitations and Future Directions

As mentioned above, an inadmissible solution was produced for the multivariate linear growth curve model assessing the association between growth in novelty seeking and anxiety. Additional time point measures, or a larger sample, may have avoided this issue (Wothke, 1993). Additional time point measures would have also allowed for the modeling of quadratic growth.

Another limitation of note is that while a significant relationship between two slope factors in a multivariate linear growth curve model indicates that two growth trajectories are related, it cannot tell us which variable causes the other. It is quite possible that increased novelty seeking results in better mental health. However, it is also possible that improvements in mental health cause an increase in novelty seeking (perhaps by allowing the individual to spend less time ruminating on negative thoughts, and more time pursuing novel interests and pursuits). Future research is needed to investigate the exact direction of this relationship. Future qualitative research may also be useful in exploring these relationships through asking participants about their experiments with lockdown and the pandemic more generally.

A third limitation is the nature of the sample. Although university students are future employees, they are not full-time workers yet. Accordingly, the results of this study should not be taken to reflect the experiences of full-time Chinese workers. Undoubtedly, there are likely some groups, such as employees who lost their jobs as a result of the lockdown (and the COVID-19 crisis more generally) who may have experienced significantly more distress during this period. However, given that 38.3 million people are currently enrolled in higher education in China (Ministry of Education of China, 2019) and that more than 80% of Chinese university students have part-time employment (Gu, 2018; Wen and Huang, 2019), the findings of the current study add value to the field of organizational psychology by demonstrating novelty seeking’s possible positive effect on mental health among university students in the time of COVID-19.

## Conclusion

The current longitudinal study found growth trajectories in which the stress, anxiety, and depression of Chinese university students decreased from prior to, to during, COVID-19 lockdown, before somewhat increasing in the post-lockdown period. Novelty seeking displayed the opposite pattern of growth. Importantly, the growth trajectory for novelty seeking was associated with the growth trajectories for stress, anxiety, and depression. These findings are of interest because: (1) they challenge the notion that pandemic lockdown is inherently detrimental to mental health, and (2) they highlight the potential protective role of novelty seeking when responding to crises.

## Data Availability Statement

The datasets presented in this study can be found in online repositories. The names of the repository/repositories and accession number(s) can be found below: The datasets generated for this study can be found in the JCU Tropical Data Hub (https://doi.org/10.25903/5ef1558919e24).

## Ethics Statement

The studies involving human participants were reviewed and approved by the Human Research Ethics Committee of the Department of Social Work, Foshan University, China (Ref. 2019001). The patients/participants provided their written informed consent to participate in this study.

## Author Contributions

WL contributed substantially to the conception of the study, design of the analysis, and writing the major portion of the manuscript. HY contributed substantially to the research design and data collection. DM contributed substantially to organizing and conducting statistical analysis, interpreting the results, and drafting the manuscript. FY contributed to the design of the study and acquisition of data. CR contributed to drafting the manuscript and providing critical revision of the manuscript. All authors contributed to the article and approved the submitted version.

## Conflict of Interest

The authors declare that the research was conducted in the absence of any commercial or financial relationships that could be construed as a potential conflict of interest.
